# Interhospital transport of critically ill patients: experiences and challenges, a qualitative study

**DOI:** 10.1186/s13049-019-0604-8

**Published:** 2019-03-04

**Authors:** Helge Eiding, Ulf E. Kongsgaard, Anne-Cathrine Braarud

**Affiliations:** 1 Division of Emergencies and Critical Care, Oslo University Hospital, Oslo, Norway; 2 The Norwegian Air Ambulance Foundation, Drøbak, Norway; 3 Medical Faculty, University of Oslo, Oslo, Norway

**Keywords:** Inter hospital, Patient safety, Patient transfer, Ambulance transport, Critically ill, Intensive care, Standard care

## Abstract

**Background:**

No consensus based national standard for interhospital transports of critically ill patients exists in Norway. The local hospitals are responsible for funding, organizing and performing these transports, resulting in potentially different level of care for the critically ill patients depending on local hospital resources and not the level of severity in the patient’s condition. The aim of this study was to examine how these transports are executed and to discover challenges during transports and potentials of improvement.

**Methods:**

A qualitative study with 20 semi-structured interviews of doctors, nurses and ambulance personnel representing a wide range in experience and formal education, reflecting the different compositions of crews performing interhospital transports was conducted. A systematic text condensation of the interviews was performed to describe personal experiences and values.

**Results:**

Few interviewees reported special adverse events when asked. Instead they chose to describe more general characteristics of the working environment, their own positive emotions or fears and the strengths and weaknesses of the organizational system. The prehospital working environment was described as different from the in-hospital environment. The personnel experienced being on their own during transports, lack of procedures and checklists and often no systematic education or demanded preparedness for participating. The resident doctors described pressure from elderly colleagues to participate in the transports. At the same time, all interviewees reported a self-interest in participating in these transports.

**Conclusions:**

Safe interhospital transports of the critically ill patients are challenged by the characteristics of the out of hospital environment. The transports are described as potentially unsafe for both patients and personnel. Systematic education is warranted, highlighting the use of checklists and special educational programs in prehospital critical care medicine. The strong personal interest to participate in these transports may serve as a barrier against changing todays system.

To ensure the right level of competence and safety for each unique patient, it is imperative to standardize the interhospital transports on a national level, built on consensus from experienced prehospital personnel.

**Trial registration:**

The trial is approved and registered by the local representative for the Norwegian Data Protection Authority as trial 13–7751.

**Electronic supplementary material:**

The online version of this article (10.1186/s13049-019-0604-8) contains supplementary material, which is available to authorized users.

## Background

There is an increasing need for interhospital transport of critically ill patients. This demand is a consequence of the specialization and regionalization that is intended to improve outcomes within intensive care [1, 2].

Interhospital transport of critically ill patients may be needed if additional technical or medical care is not available at the patient’s location. Norwegian standards on how to perform anesthesia [3] and in-hospital critical care [4] exist, but there are no national standards for interhospital transport. According to The Norwegian Patient Safety Program [5], the hospital trusts are responsible for establishing and implementing guidelines and standards for patient care. The hospital trusts are responsible for both organizing and funding interhospital transports according to the Specialized Health Services Act [6].

Furthermore, there are no defined national requirements regarding the medical or technical equipment or on the education or clinical experience of personnel accompanying the transport of critically ill patients. There are four main compositions of crews performing these transports in Norway; regular ambulances with emergency medical technicians (EMT), temporary staffed ambulances with either additional nurses or residents in anesthesiology and specialized transport units with experienced anesthesiologists [7]. In Norway it is in general the anesthesiologist who staff the specialized transport units, making it rare for other specialists to participate.

Knowing that these transports are expensive, logistically challenging, and high-risk in regard to adverse events [8], the issues of organization, patient safety and quality of interhospital transport must be addressed [9]. Thus, to learn more about how these intensive care transports are performed and experienced by the crew members, in-depth interviews with healthcare personnel who perform interhospital transports of critically ill patients on a regular basis was conducted.

## Methods

A total of 20 unique interviews were conducted from June 2013 to September 2014. For a description of study subject, see Table 1.

**Table 1:** Description of study subjects

Occupation	Label	N	F	Age	Experience in years
EMT	A	3	0	38–52	11–20
Nurse					
General	IA	2	2	36–42	16–18
Anesthetist	AS	3	2	57–58	20–32
Intensive care	IS	3	3	41–53	9–13
MD					
Resident, anesthesiology	L	6	3	31–40	0.5–3.5
Staff anesthesiologist	AL	3	1	45–58	20–24
Total		20	11	31–58	0.5–32

N; numbers, F; female, EMT; Emergency Medical Technicians

### Inclusion criteria

Emergency medical technicians, nurses, residents and staff anesthesiologists who had experience transporting patients in need of intensive care between hospitals were eligible for inclusion. Personnel from four different hospital trusts run by the South-Eastern Norway Regional Health Authority was included to cover the four main different compositions of crews and accompanying personnel in intensive care transports. The local heads of the Ambulance Service, Department of Anesthesiology and Air Ambulance Department were asked to find available interview subjects and set up appointments for the interviews.

### Research ethics

The study was approved by the local representative for the Norwegian Data Protection Authority. In addition, approval from the local representatives of the included organizations (EMTs, nurses and physicians) and the local leaders of each hospital trust was obtained.

All study subjects received written and verbal information about the major objective of the study, i.e., to “collect personal experience from interhospital transports of critically ill patients”. The right to withdraw from the study at any point during or after the interview was emphasized. All study subjects signed a written consent for participation prior to the interview.

All the invited study subjects agreed to participate when they were asked and no one have in retrospect asked to be withdrawn from the study.

### Data collection instrument

After defining the major objectives of the study to the interviewees, a semistructured interview guide, divided into two main parts, was used. All interviews started with the first part regarding the participant’s earlier experience of any specific patient transport leaving a particular impression; “The special transport”. The interviews were then shifted towards the second part containing more general topics in order to explore different aspects of the interhospital transport of critically ill patients; “Transports in general”. Several alternative sub-questions were set up (Additional file 1: Interview guide). As the interview was semistructured, there were no set alternative answers.

### Data collection

The interviews took place during regular working hours in the ambulance or in hospital quarters at each local hospital trust to ensure that the surroundings were safe and well known. The same two interviewers performed all the interviews and had separate and predetermined roles, thus ensuring uniformity among all the interviews. Both interviewers were specialists in anesthesiology and had several years of clinical experience in prehospital services, including transporting critically ill patients. One interviewer was serving as the Medical Director of the Oslo University Hospital Ambulance Service, and the other was working as an anesthesiologist both in and out of the hospital. It was emphasized that the interviewers did not work on commission from the local hospital leaders.

All interviews were recorded, and two separate recorders were used to ensure backup. The interview was always conducted by the same interviewer. The second interviewer contributed by asking supplementary questions for clarification or more in-depth questioning.

After each separate interview, the interviewers compared the information achieved from the interview with already existing data from previous interviews using a constant comparative method [10]. The interviewers then terminated further interviews at each study site when they felt saturation was achieved, i.e. when no additional disclosure of new topics or perspectives were discovered during the interviews.

All the interviews were transcribed verbatim into written accounts by personnel familiar with medical writing who proclaimed confidentiality. The transcripts were then stored in a secure server.

### Data analysis

The data analysis was performed according to Malterud’s “Systematic text condensation” [11] as follows:

#### Total impression in the bird eye perspective

Both interviewers separately read all interviews to obtain a general sense of the interviews and to intuitively identify temporary themes that drew the attention of the reader. These temporary themes may well be separate from the main themes of the interview guide.

#### Identifying and sorting meaning units – From themes to codes

The written interviews were then read with the aim of identifying “meaning units”, with a meaning unit covering one or more of the identified temporary themes from step one. This could be text fragments (quotations) containing some information about their personal experience from the interhospital transport of critically ill patients. The meaning units that related to the temporary themes or represented different aspects of the same theme were then organized into code groups. The codes were developed and adjusted several times, ending up with four code groups. The quotations were labeled with letters representing different professions (Table 1) and numbers representing each unique contributor.

#### Condensation – From code to meaning

The interviewers then collectively discussed and sorted all 108 of the identified meaning units into subgroups. All subgroups should mediate the essence from several stories, not only a chain of separate descriptions. (Additional file 2: Meaning units sorted into topics). Eighteen subgroups of meaning units were identified (Table 2).

**Table 2:** Code groups and subgroups

Code group	Organization and education
Subgroups	Hospital organization and draining of resources
	Clinical guidelines and checklist
	Inner checklist
	Training and preparedness for intensive care transports
	Learning by doing and learning from others
Code group	The out-of-hospital environment
Subgroups	Concern for the out-of-hospital transport of critically ill patients in general
	Comparing out-of-hospital work to in-hospital work
	Time consuming
	Patient information report
	Creating margins
Code group	Personal attitudes
Subgroups	Self-interest
	Lack of worry
	Relying on chance
	Being a hostage
Code group	System attitudes
Subgroups	To call for help and collegial assistance
	Being forced out of the comfort zone
	Patient safety awareness
	Reporting on adverse events

#### Synthesizing – From condensation to description and concepts

The meaning units were synthesized into analytic text, choosing quotes to represent or complement the analytical text describing the interhospital transport of critically ill patients.

## Results

### Organization and education

#### Hospital organization and draining of personnel resources

Several interviewees reported suboptimal in-house staffing when a patient needed transport, thus draining the hospital of medical personnel. One of the interviewees reported a discrepancy between planned staffing and actual staffing pertaining to out-of-hospital transports. Another interviewee described a lack of physical resources, such as emergency ambulances.

… of course, officially we have someone who is on call for transport, but it’s not always we have the staff for this … (AS1 p15).

#### Clinical guidelines and checklists

Some interviewees reported a lack of clinical guidelines for interhospital patient transport; others reported having problems finding these guidelines in the local patient safety system. The different professions had various opinions of the importance of clinical guidelines and checklists. The doctors described an interest in guidelines but revealed difficulties in finding these when needed. Other interviewees reported that guidelines existed but that they were neither fully implemented nor mandatory. None of the interviewees mentioned checklists spontaneously. When asked directly about checklists, many interviewees felt that checklists were mostly lacking or they did not know whether they existed. Some interviewees did not see the importance of having checklists.

… no, we don’t have a system in place … so it depends on the person … (AL2 p4I).

… checklists would of course be a big support … then I would’ve perhaps felt safer at an earlier stage … (IS3 p11).

#### Inner checklist

Several nurses and doctors claimed to use their own “checklists”, which they described as a “personal mental list” that they remembered. These personal checklists were not written or shared between personnel.

… there is no actual checklist, we should definitely have this, but we don’t. I have my own order of things, I kind of have my own checklist, in my head … (L2 p5).

… the safety routines you have, they are sort of in your spine … you don’t always feel like you are in control … (AS3 p17).

#### Training and preparedness for intensive care transports

All professions agreed on the necessity of specific training, both medical and technical, for performing these transports. Many interviewees felt they were expected to participate in the interhospital transport of critically ill patients despite lack of training. Several interviewees described the possibility of supervision as a one-time-only experience. Many of the doctors were concerned with lack of practice and called for more competence and experience to be able to recognize and handle potentially dangerous situations during transport. Several of the interviewees disclosed having performed intensive care transports alone without having had any experience performing intensive care treatment.

… one of the criteria for success is that the doctors shouldn’t just come along as a support without them knowing the equipment … I think it would be much better if you knew the equipment yourself as well … especially when things start to fall apart … (AL1 p10).

… not truly … not like proper scheduled training, it was more like an experienced nurse that kind of said it would be a good idea, to take this or that along with you. That was kind of the training, not the way you would think training would be … (IS3 p5).

#### Learning by doing and learning from others

After a number of transports, personnel are expected to gain experience on their own, which is described by many as “learning by doing”. They described themselves as better qualified and able to complete a safer transport after having had their own experiences. Some of the interviewees had suggestions on how the education should be implemented as a whole. They emphasized the importance of learning from adverse events experienced by colleagues. Some interviewees gave examples of good arenas for learning from others, such as short courses and meetings. One doctor suggested simulation and a more systematic education.

… what’s important is passing on your experience to others you know, not everyone has to make the same mistake to discover that something was stupid … (AL1 p9).

### The out-of-hospital environment

#### Concerns for the out-of-hospital transport of critically ill patients in general

All participants described many factors of insecurity that potentially add to a stressful workload during these transports. They all felt they were on their own regarding decision-making or emphasized the load of working on your own. Many of the interviewees expressed that during the mission, they wished for the transport to be over as soon as possible. One of the junior doctors described how working alone away from the safety of the hospital makes both themselves and the patient vulnerable.

… many of these patients are literally dying all the time … (IA2 p15).

#### Comparing out-of-hospital work to in-hospital work

Regardless of their education or experience, all the participants emphasized the striking difference between working in and out of the hospital. The out-of-hospital challenges were described as diverse, from many small hindrances sometimes adding up to the extreme stress of feeling completely alone with a deteriorating critically ill patient. They described that patients were more unstable when transported. The patients’ physiology often deteriorated during transport, making even a short transport with a stable patient potentially challenging. The parameters of medical equipment were described as less precise and sometimes not even possible to collect during transport. For these reasons, the out-of-hospital monitoring often had to be more extensive than in-hospital monitoring.

… they (the hospital staff) don’t quite understand the fact that you have much less resources, you don’t have any backup … you can’t take a break, you have to sort out basic things like the oxygen supply and things like that … they think it is the same thing (out-of-hospital and in-hospital) but it is two very different places … (AL3 p6).

#### Time consuming

Interhospital ambulance transports were described as time consuming. Several interviewees emphasized the discrepancy between the time spent on the road and the total time of the mission. They described this by how time consuming it is to prepare and stabilize the patient, collect patient information, and adjust medication and medical equipment prior to transport. One of the interview subjects even described having to compromise between the use of time and how many safety measures would be necessary.

#### Patient information report

Several of the doctors were worried that vital information regarding the patients was lost during handovers both pre- and post-transport. Sometimes, this loss of information was due to a lack of attention from the receiving personnel. Some interviewees felt that the hospital personnel wanted to be relieved of the patient responsibility as soon as possible, thus under-communicating vital information and not understanding the importance of this information for out-of-hospital personnel.

… I do notice … that when you handover and people don’t listen to you, it feels as though the message shrinks, and you may forget to say things you thought were important … (AL2 p5).

#### Creating margins

The experienced anesthesiologists spontaneously emphasized the importance of “creating margins” to secure safe patient transport. This topic was not mentioned by any other working group. “Creating margins” included everything from details such as securing intravenous lines and pharmacological relaxation of the patient to stabilizing and even changing the treatment of the patient prior to transport. They explained “creating margins” as working systematically to reduce or even eliminate the risks during transport. One of the respondents repeatedly used the expression “ensure” in order to explain the way of working outside of the hospital. Another respondent compared the preparations for adverse events during transports to athletes’ preparations prior to a competition.

… the transport medicine, partly the emergency medicine, but especially the transport medicine, is about being systematic and creating margins … (AL1 p17).

### Personal attitudes

#### Self-interest

Most of the participants expressed some degree of positive self-interest in participating in prehospital work in general and especially in the interhospital transport of critically ill patients. Transporting these patients was often explained as a good way to learn emergency medicine. Several of the doctors experienced being forced to step up to the challenge and thereby learn decision-making. Some subjects described the out-of-hospital experience as a kind of entertainment and something covering the need for a “break” from everyday work.

… it is kind of like a fearful joy, because, it is actually kind of fun in a way … (L3 p2).

… when people suddenly show trust toward you … I do enjoy that … (IA2 p6).

#### Lack of worry

Several of the nurses spontaneously described these transports as easy to perform and felt no reason to worry. Two of the nurses felt very safe in believing the patients to be well-prepared and stabilized prior to each transport. They described these transports as being “safe and easy”.

… the intensive care transfers are actually often the safest ones, because the patients there are properly prepared, even though they are very ill, they still, in a way, have all the right monitoring and they have secured airways … (AS3 p5).

#### Relying on chance

Some of the personnel considered themselves to be lucky when adverse events and patient deterioration were avoided during transports, while others expressed the hope of avoiding challenging clinical situations in future transports.

… now, I have actually been very lucky, as in the fact that the patients I have transferred have been stable, and it has sort of all gone well … (IS1 p5).

#### Being a hostage

One staff anesthesiologist expressed the feeling of being a hostage during transport; another regarded his level of competence to be a risk of substandard treatment of the patient and, at the same time, inadequate care of himself as a colleague, being expected to participate in these transports.

… we all felt that it was unsafe (intensive care transfers as a junior doctor), we all sat there and held the tube, hadn’t even been inside an ambulance before … and the consultants didn’t want to go, so I remember that we were the ones who were sent, we didn’t even know how to intubate, so you kind of became a hostage, like a substitute for a bad fixation of the tube, that was kind of what is was like … when I think about it now I think it was truly badly done … they (the consultants) didn’t dare, they knew it could be dangerous you know … and we were slightly too stupid to understand it … (AL3 p21).

### System attitudes

#### To call for help and collegial assistance

Most of the interviewees explained how inexperienced personnel are offered the opportunity to call a colleague when in need during transports to compensate for their lack of skills and knowledge. This offer was described as a safety measure for both doctors and nurses during transports. Some of the inexperienced doctors described it as comforting to know they could call someone to discuss ongoing treatment or patient deterioration.

… you should talk to others, you should “call a friend” if you are in need, it is possible to call others both in the hospital where you are picking up but also definitely at the receiving hospital, you could also call others who are experienced with patient transports … (AL1 p15).

#### Being forced out of the comfort zone

All the residents gave the impression of being under pressure. They felt they were expected to work outside of the hospital even though they felt incompetent due to a lack of experience and education.

… I do remember, in the beginning I felt forced … and I did say that I was not comfortable with this, and that I truly didn’t want to do this, but few seemed to truly understand … (L1 p19).

… so I do think it is a stress factor just being at call actually, the fact that you could get sent out, and be completely on your own … (L2 p12).

#### Patient safety awareness

All the interviewees discussed patient safety when asked about the topic specifically. The experienced anesthesiologists were particularly concerned with this and shared many self-experienced stories concerning patient safety in general. One of the subjects used the resemblance to aviation safety when evaluating patient safety. Formalized education, medical treatment protocols, learning from others and checklists were also mentioned as parts of a patient safety system.

… the aviation industry stopped measuring their quality by the number of accidents ages ago because they (accidents) are very rare … so we cannot use the fact that someone died during transport or not … luckily, not many die during the transport, and that is a good thing, but that doesn’t mean that the rest of it is any good … (AL1 p31).

#### Reporting on adverse events

The interviewees were not asked specific questions about reporting adverse events, but several still mentioned the topic. Lack of oxygen supply and battery capacity during transport was pointed out as well-known events for the novice personnel, but no specific transports where this had occurred was actually brought up. Some interviewees had experienced that adverse events were not necessarily reported and proposed several explanations for this. Some participants described reporting adverse events as uncomfortable, while others described reporting these events as time consuming.

… a lot of things do happen that should have been reported as an adverse event, but it does take time to actually sit down and write in Synergy (registration system for adverse events) …

some people may even think this is uncomfortable to do as well, especially if colleagues are involved … (AS1 p19).

## Discussion

To learn more about personal experiences, episodes, values and interaction, Malterud recommends the use of qualitative methods [12]. In-depth interviews were chosen, being a method recommended for revealing personal experiences and ethical values. The interviewers had personal field experience from prehospital patient transports and from former use of in depth interviews in research. They both had a theoretical background that supported their choice of topics for the interviews. To enhance the reliability and validity of the interviews, the conceptual framework, communication process and analysis of the text was addressed and thoroughly discussed in the planning of the study.

Knowing that the interhospital transport of critically ill patients is organized in mainly four different ways, individuals from four specific hospital trusts were eligible for interviews. In qualitative research a limited number of informants can be chosen. The strategic sample size of this study was not decided in forehand but decided to be achieved when the interviews presented a diversity in information, contradictions and paradoxes that could enlighten the research topics. To ensure this, stepwise recruitment and analysis of the richness of information gained decided the number and type of interviewee objects. The participants had a wide range of experience and represented all working organizations (EMTs, nurses and physicians), thus ensuring a maximum variation sampling. In the resident group, a larger number of interviewees were chosen to cover a variation in personal experience with transports. [13]

There is no official registration of the number of transports performed by each personnel, hence, as a surrogate, the experience in years represents the interviewees experience of interhospital transports.

In our experience, the interviewees were distinct, and the participants individually brought up topics and themes that provided an accurate description of their experiences. All respondents spontaneously presented both contradictions and paradoxes, indicating reflections at a personal level. Their experiences were often paired with suggestions on how the system could provide better patient safety and a safer working environment. We believe these spontaneous themes and experiences give a true description of the different organizational systems for intensive care transports and not just a socially acceptable description of how the service should be organized.

According to the interview guide, the interviewees were initially asked to share an experience from a transport that made a special impression, followed by their impression of transports in general. We were anticipating stories containing adverse events and even dramatic outcomes [14], but surprisingly few of the interviewees gave any details of adverse events except general comments on running out of battery or oxygen.

The interviewees presented working environments with a striking lack of educational systems, procedures and checklists for the transport of critically ill patients. All participants described having a great self-interest in participating in these transports. The residents emphasized this despite worries about own personal suitability for the work and lack of specific education. Expectations from the consultants on duty were sensed as a pressure to participate, especially by the interns. The combination of self-interest and sensed expectations can potentially facilitate an unsafe environment for both the personnel and this vulnerable patient group.

The out-of-hospital treatment of critically ill patients should be of the same quality and safety level as in-hospital treatment [15], but there is no consensus on a standard for the transfer of intensive care patients in Norway. These transports are performed under different routines, with different equipment, with few if any checklists and by different professions with variable experience from different hospital trusts run by the South-Eastern Norway Regional Health Authority.

Many of these transports were described as challenging for the local hospital resources on call, often resulting in the least experienced personnel being forced to participate in these transports, only offered the opportunity to call a senior doctor during transports as a safety measure. In reality we believe this safety measure only to be a method of verbal moral support, demanding the inexperienced personnel en route to understand when to call for help and when to sort out events alone. This may contribute to a false sense of safety for the personnel and thus lower the threshold of participation in the transport of complicated or unstable patients. The inexperienced crew member even described the feeling of being a hostage during these transports due to their felt lack of competence and the feeling of being on their own.

The fact that none of the interviewees discussed checklists until they were asked directly is surprising, knowing that the use of checklists influences important working processes in surgery by reducing complication rates and the length of hospital stay [16], and during transports, is associated with a reduction of adverse events [15]. The creation of personal inner checklists based on self-experienced adverse events and needs derived from prior transports, in our opinion shows that the performance as a whole was left to chance during these transports.

Transporting the critically ill patient is more time consuming than just the transport itself, often demanding stabilization and even a change of treatment prior to leaving the hospital. This point was emphasized only by the experienced doctors, describing the importance of “creating margins” for the patient by spending more time initially to facilitate a safer transport. [17]

The out-of-hospital environment was described as very different from the in-hospital environment by most of the participant. The more experienced personnel emphasized not being able to trust the equipment for surveillance and being alone or deprived of resources while caring for unstable patients out-of-hospital. The in-hospital colleagues are often perceived as ignorant of the out-of-hospital challenges, independent of, and even despite of, their in-hospital skills [18]. This ignorance may result in the underreporting of challenges in patient treatment during the handover and thereby increase the potential challenges during transports.

The interviews revealed how participants with increasing experience became increasingly more aware of safety issues and the necessity to prevent adverse events. The different levels of reported concern for performing interhospital transports of critically ill patients may be a Dunning-Kruger-effect [19], which suggests that those with little experience, as a result of ignorance, accept the risk of transport on behalf of the patient. This Dunning-Kruger-effect, combined with the large degree of self-interest in performing these transports focusing not on the patient but on fail-and-learn based self-education, might explain the willingness to accept the system as it is.

The participants also identified hindrances in reporting adverse events, such as time consuming systems and the fear of revealing the events to colleagues thus resulting in the underreporting of adverse events.

The skills of working with critically ill patients outside of the hospital must be systematically learned, as in all medical disciplines, including knowledge of how to create margins to transport the patient safely. We find that this provides a potential for structured education and learning from others instead of requiring that all personnel have to experience all the pitfalls of out-of-hospital transports on their own.

Having a national standard could guide the level of competence needed depending on the patient’s condition, similar to in-hospital critical care, and thereby define the level of education needed for the personnel who accompany critically ill patients. [18]

## Conclusion

Interhospital transport of critically ill patients was described as time consuming, draining of local hospital resources, logistically challenging and potentially unsafe for the patients.

Most of the personnel warranted systematic education and wanted to learn from more experienced personnel in general and from previous adverse events in particular. Patient safety issues, the use of checklists and special educational programs were highlighted as areas for improvement.

The interviews revealed how the out-of-hospital environment demands special considerations concerning education and system planning. The strong personal interest in participating in the transport of critically ill patients may serve as a barrier against the changes of today’s system.

The time for standardizing the transport of critically ill patients is ripe. This standardization should be on a national level and include directions for improving the education and competence of accompanying health personnel, procedures and checklists, and tools for safe handover and decision-making both before and during transport. The national transport standard should be built on a consensus from experienced personnel to ensure an appropriate level of demanded competence for each unique patient, thereby securing patient safety.

## Additional files

Additional file 1: Interview guide. (DOCX 16 kb)

Additional file 2: Meaning units sorted into topics. (DOCX 26 kb)
