# Supplementary material for: Interhospital transport of critically ill patients: experiences and challenges, a qualitative study
Source: Scand J Trauma Resusc Emerg Med. 2019 Mar 4;27:27. doi: 10.1186/s13049-019-0604-8 (PMC6399939; doi:10.1186/s13049-019-0604-8)
Supplement: Supplementary file 1 — Interview guide. (DOCX 16 kb) [file 13049_2019_604_MOESM1_ESM.docx]

# Additional file 1

# Interview guide

**Theme / structure for interview**

EMT

Nurse

MD (resident / anesthesiologist)

**Introduction / preparation** (less than half a page)

Written information is sent in advance

Let the candidate **read** the content: **ask if something is unclear.**

**Clarify the following:**

- **You can stop at any time;**
- **Anonymity (get a respondent number);**
- **You have the right to have the interview deleted (you cannot change parts of the interview).**

**The interview**

**Formalities**

What is **your formal or highest level of education**?

**For how long have you been** working in this profession?
Where do you work **now**? (for the tape)

How **old** are you?

**This is what we are going to talk about:**

In this conversation, we want **to hear about your experiences and your thoughts regarding the transport** of intensive care patients between hospitals.

By **intensive care patients, we mean patients who are being transported from, to or between** intensive care departments because they need intensive care monitoring and treatment (for example, intubation or vasoactive treatment) healthcare that they cannot get in a normal hospital ward.

**Two types of intensive care transport:**

To make it easier, we **put patient transports into two different categories: planned transports, where we have some time to arrange, plan and order** the transportation and choose the best qualified health professionals, and **ad hoc transports, which must be arranged very quickly and during duty hours** because the patient needs a quick transport in order to receive better help at another hospital.

1. **The special transport:**
2. Have you ever attended these transports? One or several?

Do you remember a transport like this? Or

1. **Did any of these transports leave a special impression on you?**

Do you remember the transport…?

Would you call it a planned transport or an ad hoc transport?

1. Can you **tell more about this transport, the way you remember** it?

Were you **the only** **accompanying personnel?** (The treatment rooms?)

How did you **experience the interaction?**

Can you tell me some more about it?

(Could you choose how the transport was carried out?)

What do you **feel about the transport**?

Do I **understand you correctly** when you say that…?

How did the transport go?

Did you feel **that it was safe?**

1. **Transports in general:**
2. Have **any transports felt unsafe?** (Unsafe for the patient?)
3. Have you ever experienced **failure or situations** that could lead to **consequences for the patient?**

Can you **say something about what happened?**

(Try to explain how it proceeded and the consequence for the patient.)

How was **the communication?**

Deficiencies with the car? Medical or technical?

Do you feel **that you have had enough knowledge and education** during the transports?

3) What **do you think is necessary** in order to provide proper assistance during intensive care transports?

Can you say something general about the transports? Something about the **preparations, equipment and personnel.**

Scoring of the patient

Mandatory personnel

Mandatory checkout

4) Do you have any **experience with these transports from anywhere else** in Norway or abroad?

**The final part**

Do you think **this interview went well?**

Is **there anything you feel you didn´t get the chance to say** or something **you want to add** before we finish this interview?
