# Supplementary material for: Interhospital transport of critically ill patients: experiences and challenges, a qualitative study
Source: Scand J Trauma Resusc Emerg Med. 2019 Mar 4;27:27. doi: 10.1186/s13049-019-0604-8 (PMC6399939; doi:10.1186/s13049-019-0604-8)
Supplement: Supplementary file 2 — Meaning units sorted into topics. (DOCX 26 kb) [file 13049_2019_604_MOESM2_ESM.docx]

# Additional file 2

**Meaning units sorted into topics**

Meaning units Number mentioned relevant to the topic (Number of interviewees mentioning meaning unit)

**Code group Organization and education**

Subgroup *Hospital organization and draining of personnel resources*

No 15

Meaning units

*Resource* AL L 1(1) AS 3(2) IS IA 1(1) A 2(1)

*Readiness* AL L 1(1) AS 1(1) IS IA A 1(1)

*Enough staff*  AL L 1(1) AS IS IA A

*Personnel* AL L 1(1) AS IS IA A 12

Subgroup *Clinical guidelines and checklist*

No 9

Meaning units

*Procedures* AL 2(2) L 5(3) AS IS 5(3) IA A 6(2)

*Systems* AL 19(3) L 12(5) AS 3(1) IS 1(1) IA 3(1) A 2(2)

*Recipe* AL 1(1) L AS IS IA A

No 1

Meaning units

*Checklist* AL 7(2) L 6(2) AS IS 3(2) IA 1(1) A 76

Subgroup *Inner checklist*

No 18

Meaning units

*Checklist (inner)* AL 2(1) L 6(4) AS 2(2) IS IA A

*Control* AL 7(2) L 6(3) AS 3(2) IS 4(3) IA A 5(3)

*Thorough* AL 2(1) L AS IS IA A

*Ensure* AL 1(1) L AS IS 1(1) IA 2(1) A

*Want* AL L 1(1) AS 3(1) IS 1(1) IA A 2(2) 48

Subgroup *Training and preparedness for intensive care transports*

No 25

Meaning units

*Learning* AL 7(3) L 31(6) AS 4(3) IS 4(2) IA 11(2) A

*Education* AL L AS 2(2) IS 1(1) IA A

*Be introduced* AL 1(1) L 1(1) AS IS IA A 1(1)

*Advise* AL 3(1) L 1(1) AS IS 4(3) IA A 71

Subgroup *Learning by doing and learning from others*

No 31

No 36

Meaning units

*Experience* AL 6(3) L 16(5) AS 6(3) IS 8(3) IA 2(1) A 2(1)

*Newcomer* AL 2(1) L 8(3) AS 1(1) IS IA 2(1) A

*More than before* AL L 2(2) AS IS IA A

*Share events* AL L 1(1) AS IS 1(1) IA A

*Truly felt* AL 1(1) L AS IS IA A 58

*Pass on experience* AL L 1(1) AS IS IA A

*Cases* AL L AS 1(1) IS IA A

*Share experience* AL 1(1) L 2(2) AS IS IA A

*Tips* AL 6(1) L 5(2) AS IS IA A 16

**Code group The out-of-hospital environment**

Subgroup *Concern for the out-of-hospital transport of critically ill patients in general*

No 32

Meaning units

*Fit* AL L 1(1) AS IS IA A

*Secure* AL L 6(5) AS 8(2) IS 7(2) IA A 1(1)

*Watch* AL 3(2) L AS IS 1(1) IA A 1(1)

*Alone* AL 6(2) L 12(5) AS 5(2) IS 3(2) IA 3(1) A

*Hope* AL L AS 1(1) IS 1(1) IA A 1(1)

*Catastrophic* AL L AS 1(1) IS IA A

*Unrest* AL 1(1) L AS IS IA 2(1) A

*Afraid* AL 5(2) L AS 1(1) IS IA 2(1) A

*Danger* AL 1(1) L AS IS IA A

*Scary* AL 1(1) L AS 1(1) IS IA A 1(1)

*Paranoid* AL 4(1) L AS IS IA A

*Fail all the time* AL 1(1) L AS IS IA A 81

Subgroup *Comparing out-of-hospital work to in-hospital work*

No 8

No 14

Meaning units

*(Un)stable* AL 5(2) L 24(6) AS 8(3) IS 9(3) IA 5(1) A 7(2)

*Transportable* AL L AS 2(2) IS IA A

*Medical history* AL L 1(1) AS IS IA A 1(1)

*Brief*  AL 7(2) L 1(1) AS 5(2) IS 2(1) IA 3(2) A 3(2)

*Dialogue* AL 1(1) L 1(1) AS IS IA A 85

No 31

Meaning units

*Ventilator* AL 31(3) L 10(4) AS 7(2) IS 12(3) IA 10(2) A 3(1)

*Equipment* AL 31(2) L 4(1) AS 11(3) IS 11(3) IA 19(2) A 17(3)

*Machines* AL 7(2) L AS IS IA A

*Battery* AL 10(2) L 3(1) AS 1(1) IS 2(2) IA A 3(1)

*Charging* AL 14(2) L 2(2) AS IS 1(1) IA A 1(1) 204

No 32

Subgroup *Time consuming*

No 13

Meaning units

*Time* AL 8(2) L 12(4) AS 2(1) IS 9(3) IA 8(2) A 9(1)

*Hour* AL L AS 2(1) IS 3(2) IA 1(1) A 9(2) 63

Subgroup *Patient information report*

No 13

Meaning units *Time / Hour*

Subgroup *Creating margins*

No 27

Meaning units

*Margin* AL 12(3) L AS IS IA A

*Bastards in the system* AL 2(1) L AS IS IA A 14

No 19

Meaning units

*Quality* AL 6 (2) L 6 (3) AS IS IA A

*Proper* AL 3(1) L 4 (1) AS IS 2(1) IA A

*Expected level* AL L 1(1) AS IS IA A

*Worst case* AL 1(1) L AS IS IA A

*Gamble* AL 2(1) L AS IS IA A 1(1)

*Trust* AL 3(2) L 1(1) AS IS 1(1) IA 1(1) A 1(1)

*Risk* AL 4(3) L AS IS 2(1) IA A 1(1) 39

No 8

Meaning units

*Work out of hospital* AL L AS 2(1) IS IA 2(2) A 1(1)

*In the ambulance* AL 1(1) L 4(4) AS 5(3) IS 7(2) IA A 1(1)

*Underway* AL 7(2) L 9(3) AS 3(1) IS 5(3) IA 3(1) A 2(2) 52

**Code group Personal attitudes**

Subgroup *Self interest*

No 20

Meaning units

*Personal* AL L 1(1) AS IS IA A

*Enjoy* AL L 2(2) AS IS 6(2) IA A

*Thrilling* AL L 3(2) AS IS IA 1(1) A

*Allowed to* AL 3(1) L AS IS IA 2(1) A

*Fun* AL 1(1) L AS IS 3(2) IA A 2(2)

*Want to* AL L 1(1) AS IS IA 1(1) A

*All right* AL L 2(1) AS 1(1) IS 1(1) IA 4(2) A

*Exciting* AL 1(1) L AS IS 1(1) IA 2(2) A

*Thrive* AL L AS 1(1) IS IA A 38

Subgroup *Lack of worry*

No 38

Meaning units

*Not felt unsafe* AL L AS 2(1) IS 2(1) IA A

*Safe* AL L 4(4) AS 3(1) IS IA A 1(1)

*Simple missions* AL L AS 1(1) IS IA A 13

Subgroup *Relying on chance*

No 42

Meaning units

*Passed easily* AL L 3(3) AS IS IA 1(1) A

*Hope* AL L 1(1) AS 1(1) IS 1(1) IA 1(1) A 1(1)

*Lucky* AL L 3(2) AS 3(1) IS 4(2) IA A 4(2) 23

Subgroup *Being a hostage*

No 33

Meaning units

*Hostage* AL 1(1) L AS IS IA A

*Shared responsibility* AL 4(2) L 4(3) AS 3(3) IS 4(2) IA 1(1) A 3(3)

*Consultant opinion* AL L 1(1) AS IS IA A

*Emergency law* AL 1(1) L AS IS IA A

*Prehospitally unexperienced* AL 1(1) L AS IS IA A

*Take charge* AL L 1(1) AS IS IA A 24

**Code group System attitudes**

Subgroup *To call for help and collegial assistance*

No 39

Meaning units

*Call* AL 3(3) L 3(3) AS 2(2) IS 2(1) IA 1(1) A 1(1) 14

Subgroup *Being forced out of the comfort zone*

No 43

Meaning units

*Cowboy* AL 1(1) L 2(2) AS 1(1) IS IA A

*Jump in* AL L 1(1) AS IS IA A

*Sort of hit and miss* AL L 1(1) AS IS IA A

*Alone* AL 3(2) L 6(4) AS 3(2) IS 3(2) IA 1(1) A 1(1)

*Stressful (and scary)* AL L 4(1) AS IS IA 1(1) A

*In the beginning...I didn`t know* AL L 1(1) AS IS IA A

*How could you* AL L 1(1) AS IS IA A

*Pressured* AL 1(1) L 2(1) AS IS IA A

*Too early* AL L 2(1) AS IS IA A

*Why shouldn´t we* AL L 1(1) AS IS IA A

*Someone has to* AL L AS IS 1(1) IA A 37

Subgroup *Patient safety awareness*

No 19

Meaning units

*Quality* AL 5(2) L 3(2) AS IS 1 1) IA 1(1) A

*Proper* AL 2(1) L 4(1) AS IS 1(1) IA A

*Arrogance* AL L 1(1) AS IS IA A

*Worst case* AL 1(1) L AS IS IA A

*Gamble* AL 1(1) L AS IS IA A 1(1)

*Trust* AL 2(2) L 1(1) AS IS 1(1) IA A

*Risk* AL 5(3) L AS IS 1(1) IA A 31

Subgroup *Reporting an adverse event*

No 34

Meaning units

*Adverse event* AL 6(2) L AS 3(1) IS IA 1(1) A

*(Potentially) serious* AL 3(2) L AS IS IA A

*Tell* AL 1(1) L AS IS IA A

*Report* AL 4(2) L AS IS IA A 18

Total 1017
